# The Incidence and Repetition of Hospital-Treated Deliberate Self Harm: Findings from the World's First National Registry

**DOI:** 10.1371/journal.pone.0031663

**Published:** 2012-02-20

**Authors:** Ivan J. Perry, Paul Corcoran, Anthony P. Fitzgerald, Helen S. Keeley, Udo Reulbach, Ella Arensman

**Affiliations:** 1 Department of Epidemiology and Public Health, University College Cork, Cork, Republic of Ireland; 2 National Suicide Research Foundation, Cork, Republic of Ireland; 3 Child and Adolescent Mental Health Services, HSE South, Mallow, County Cork, Republic of Ireland; The University of Queensland, Australia

## Abstract

**Background:**

Suicide is a significant public health issue with almost one million people dying by suicide each year worldwide. Deliberate self harm (DSH) is the single most important risk factor for suicide yet few countries have reliable data on DSH. We developed a national DSH registry in the Republic of Ireland to establish the incidence of hospital-treated DSH at national level and the spectrum and pattern of presentations with DSH and repetition.

**Methods and Findings:**

Between 2003 and 2009, the Irish National Registry of Deliberate Self Harm collected data on DSH presentations to all 40 hospital emergency departments in the country. Data were collected by trained data registration officers using standard methods of case ascertainment and definition. The Registry recorded 75,119 DSH presentations involving 48,206 individuals. The total incidence rate fell from 209 (95% CI: 205–213) per 100,000 in 2003 to 184 (95% CI: 180–189) per 100,000 in 2006 and increased again to 209 (95% CI: 204–213) per 100,000 in 2009. The most notable annual changes were successive 10% increases in the male rate in 2008 and 2009. There was significant variation by age with peak rates in women in the 15–19 year age group (620 (95% CI: 605–636) per 100,000), and in men in the 20–24 age group (427 (95% CI: 416–439) per 100,000). Repetition rates varied significantly by age, method of self harm and number of previous episodes.

**Conclusions:**

Population-based data on hospital-treated DSH represent an important index of the burden of mental illness and suicide risk in the community. The increased DSH rate in Irish men in 2008 and 2009 coincided with the advent of the economic recession in Ireland. The findings underline the need for developing effective interventions to reduce DSH repetition rates as a key priority for health systems.

## Introduction

Suicide is a significant public health issue with almost one million people dying by suicide each year worldwide, representing an annual global suicide mortality rate of 16 per 100,000 [1]. Deliberate self harm (DSH) is the single most important risk factor for suicide [2], [3]. UK studies have estimated that in the year after an act of DSH the risk of suicide is 30–50 times higher than in the general population [4], [5]. Self-inflicted injuries are the sixth leading cause of ill-health and disability worldwide [6]. In England, it is estimated that there are 220,000 hospital attendances annually due to DSH [7].

DSH frequently leads to non-fatal repetition with worldwide studies giving an estimated median risk of repetition of 16% within one year and 23% over four years of follow-up [5]. Subsequent repeated self harm often occurs within days of a DSH act [8], [9]. A UK multicentre study showed a repetition rate of 19% within one year of an index case but analysis based on all observed DSH presentations gave a much higher rate of repeated DSH (33%) [10].

Few countries worldwide have reliable data on DSH [11]. There is a broad spectrum of self harming behaviours ranging from deliberate recklessness to highly lethal attempts at suicide and only a minority of adolescents and adults who self harm presents to hospital [12], [13].

An internationally-agreed definition of DSH has been endorsed by the WHO. This definition facilitated international comparison in incidence and trends in a European multicentre study which demonstrated more than 10-fold variation in rates across more than 20 regions [14]. These incidence rates were based on single centres and reliable estimates of national rates are not available.

We have established a national DSH registry in the Republic of Ireland. The purpose of this national registry is to determine and monitor the incidence and repetition of the problem, to identify high-incidence groups and areas and to inform services and practitioners concerned with the prevention of suicidal behaviour. In the current paper, we present person-based rates of DSH at national level together with data on the spectrum and pattern of DSH presentations and risk of repetition in the Republic of Ireland over the seven-year period 2003–2009.

## Methods

### Ethics Statement

Ethical approval was granted by the National Research Ethics Committee of the Faculty of Public Health Medicine. The Ethics Committee and the Irish Data Protection Agency gave approval to the Registry operating without obtaining informed patient consent. The National Suicide Research Foundation is registered with the Data Protection Agency and complies with the Irish Data Protection Act of 1988 and the Irish Data Protection (Amendment) Act of 2003.

### Setting and coverage

The population of the Republic of Ireland increased by 12% to almost 4.5 million in the study period 2003–2009. During this period, 40 hospitals operated emergency departments (EDs) in Ireland. The number of hospitals that contributed full calendar year data to the Registry increased from 37 hospitals for 2003 to 38 for 2004–2005 and all 40 hospitals for 2006–2009.

### Definition of deliberate self harm

The Registry uses the following as its definition of deliberate self harm: ‘an act with non-fatal outcome in which an individual deliberately initiates a non-habitual behaviour, that without intervention from others will cause self harm, or deliberately ingests a substance in excess of the prescribed or generally recognised therapeutic dosage, and which is aimed at realising changes that the person desires via the actual or expected physical consequences’ [14]. This definition was derived for the WHO study and it is consistent with that used in the UK Multicentre study [7]. The definition includes acts involving varying levels of suicidal intent and various underlying motives such as loss of control, cry for help or self-punishment.

### Data items and coding

The Registry core dataset includes the following variables: patient initials recorded in an encrypted format, gender, date of birth, area of residence coded to administrative area (electoral division), date and hour of attendance at hospital, method(s) of self harm, drugs taken (if applicable), and recommended next care. The method(s) of self harm were recorded according to the Tenth Revision of the WHO's International Classification of Diseases codes for intentional injury (X60–X84). In cases where more than one method of self harm was used, all were recorded. For cases of self harm involving alcohol, the code X65 was recorded. Data items that are not routinely available in emergency department charts and records, such as suicide intent and psychiatric diagnosis, are not recorded by the Registry.

### Data collection and quality assurance

All data were collected by data registration officers who operated independently of the hospitals and followed the Registry's standardised methods of case ascertainment. In 36 hospitals, all electronic or paper-based records of attendances to the ED were checked manually by the data registration officers. In four hospitals, a two-stage process has been developed whereby a keyword search is first undertaken of the free-text field in the ED's IT system which records the reason for the presentation as completed by the triage nurse. All presentations flagged as potential cases by the keyword search are then examined by the data registration officer. From 2003 through 2005, Registry data were recorded on pre-printed forms that were optically scanned at the Registry offices with data capture using an integrated high resolution character recognition software and scanner system. From 2006, the Registry has recorded data onto laptop computers via a customised data entry and electronic transfer system.

All data registration officers receive standard training in the Registry data collection methods and procedures and attend biannual update meetings which review case definitions and related quality control issues. In 2004 and 2005, a quality control exercise was carried out whereby a pair of data registration officers each independently collected data from two hospitals for the same consecutive series of attendances to the ED. High levels of agreement were found between the data registration officers in terms of case ascertainment, with Kappa statistics of 0.97 in 2004 and 0.95 in 2005.

### Data analysis

Encrypted patient initials, date of birth and gender were the data items used to distinguish between DSH patients and to identify repeat presentations by the same patients. The Registry recorded 243 presentations in 2003–2009 lacking at least one of these data items. These cases constituted just 0.3% of all recorded cases and were excluded from all analyses presented here.

The annual incidence rate per 100,000 population was calculated for the total, male and female population (and for age-sex subgroups) based on the number of persons who presented to hospital following DSH in each calendar year. Where appropriate, direct standardisation was carried out using the European Standard Population [15]. Population data were derived at all required levels of disaggregation from the National Census 2006. Annual population estimates provided by the Irish Central Statistics Office were used for non-censal years if available at the required level of disaggregation. Exact Poisson 95% confidence intervals were calculated for the rates using StatsDirect version 2.7.7.

The year 2006 was the first year that all 40 hospital EDs in the Republic of Ireland contributed data to the Registry as in 2003 the Registry had data from 37 hospitals and in 2004–2005 there were data from 38 hospitals. Using 2006, the national incidence rates reported in the paper were estimated in three ways: based on data from all 40 hospitals (rate 1), based on data from the 37 hospitals that contributed to the Registry in 2003 (rate 2) and based on the data from the 38 hospitals that contributed to the Registry in 2004–2005 (rate 3). Relative differences were calculated for 2003 (i.e. rate 1 divided by rate 2) and 2004–2005 (rate 1 divided by rate 3). These relative differences were then applied to the rates calculated using the available 2003 and 2004–2005 data.

A repeat DSH episode was defined as re-presentation to any hospital ED due to a further act of self harm undertaken after leaving hospital following treatment in an ED for a previous act of DSH.

The repeat event analysis used was conditional risk set analysis [16], [17]. This analysis examined age, gender, method of self harm, and the number of previous self harm episodes as possible risk factors for repetition. Univariate results were presented using a series of Kaplan-Meier cumulative incidence curves. Multivariate analysis used a Cox proportional hazard model. We used a robust analysis that modified the variance of the estimates to account for lack of independence between DSH presentations by a given individual. Analyses involving the number of previous DSH presentations were confined to persons whose first observed presentation was made after 1 January 2006 (i.e. no DSH presentation in 2003–2005).

The time from the start of follow-up, 1 January 2006, until a subject's index event may reflect a subject's underlying risk of DSH. We considered a model that included an additional adjustment for this lead-in time. While increased lead-in time was associated with reduced risk of repetition, adjustment for it did not impact on the results for the factors of interest.

When the final date of the study is fixed, 31 December 2009 in this case, the potential follow-up time will depend on when a subject enters the study. Individuals with high risk of repetition would be expected to enter the study sooner and so have a longer potential follow-up time. This leads to informative censoring that can bias final estimates [18]. To address this, we limited follow-up after a self harm presentation to 12 months. Events that occurred after 12 months were censored and we only followed subjects who presented before 2009. This ensured that all subjects had the same potential follow-up of one-year and means that repetition risk measures related to repetition within 12 months. The assumption of proportional hazards was tested graphically. Analysis was carried out using Stata 11.

## Results

### Deliberate self harm 2003–2009

For the period 2003–2009, the Registry recorded 75,119 DSH presentations to hospital EDs in Ireland, involving a total of 48,206 individuals. The average annual total, male and female rate of persons presenting with self harm were 198 (95% CI: 196–200), 173 (95% CI: 171–175) and 224 (95% CI: 221–226) per 100,000, respectively. The total incidence rate fell by 2–6% annually from 2003 (209 (95% CI: 205–213) per 100,000) to 2006 (184 (95% CI: 180–189) per 100,000) and then increased by 2–6% per year, returning to 209 (95% CI: 204–213) per 100,000 in 2009. The most notable annual changes were two successive 10% increases in the male rate of DSH (from 162 (95% CI: 157–168) per 100,000 in 2007 to 179 (95% CI: 173–185) per 100,000 in 2008 and 197 (95% CI: 191–203) per 100,000 in 2009). Overall, the female rate was 29% higher than the male rate though this sex difference reduced in recent years from 38% in 2005 to just 13% in 2009.

### Gender and age

**Figure 1:**
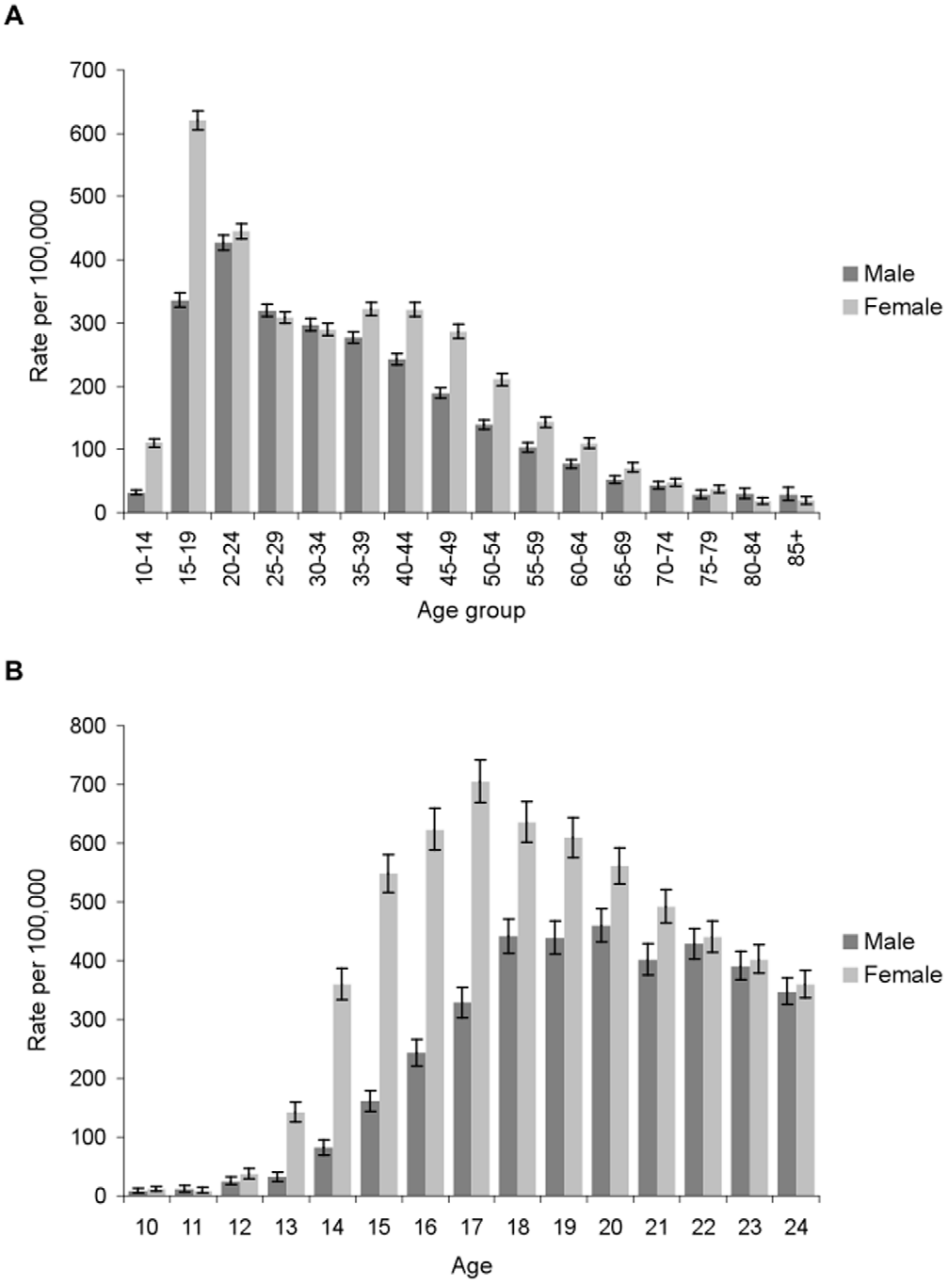
Incidence of hospital-treated deliberate self harm by five-year age-sex group (A) and by single year of age for 10–24 year-olds (B). (A) The annual rate per 100,000 population of male and female persons presenting to hospital as a result of deliberate self harm is shown by five-year age group with error bars representing the 95% confidence interval around the rate. (B) The annual rate per 100,000 population of male and female persons presenting to hospital as a result of deliberate self harm is shown by single year of age for 10–24 year-olds with error bars representing the 95% confidence interval around the rate.

There was substantial variation in the incidence of hospital-treated DSH by age for both men and women (Figure 1a), with a clear peak in 15–19 year-old women (620 (95% CI: 605–636) per 100,000), almost twice the equivalent male rate (336 (95% CI: 325–347) per 100,000). In men, the highest rate was observed in the 20–24 year age group (427 (95% CI: 416–439) per 100,000). However, this was marginally lower than the rate in 20–24 year-old women (445 (95% CI: 433–457) per 100,000). DSH was relatively rare in older people. In contrast there was an appreciable incidence of DSH in late childhood and adolescence, particularly in girls and in analyses by single year of age for the 10–24 year age group the peak rate was at age 17 in girls (705 (95% CI: 669–743) per 100,000) whereas the male rate peaked at age 20 years (460 (95% CI: 433–488) per 100,000; Figure 1b).

### Method of deliberate self harm

**Figure 2:**
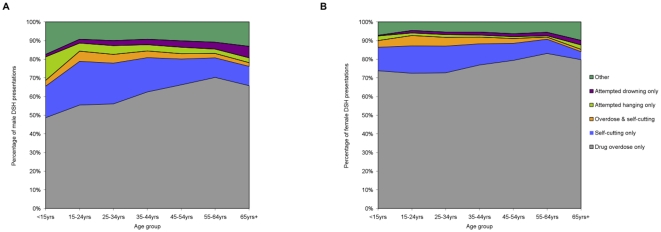
Method of deliberate self harm (DSH) involved in male (A) and female (B) presentations to hospital in Ireland. The areas illustrated in the charts represent the percentage of male and female deliberate self harm presentations involving different methods of self harm. Variation in the areas across the X-axis illustrates the variation in the method of self harm used by age.

Figure 2 shows variation by age in the method of DSH used by men and women who presented to hospital EDs for treatment. Drug overdose was by far the most common method of self harm involved. It was the sole method used in 68% of all cases and more so for women (75%) than for men (59%; P<0.001). The most common types of drugs involved in intentional overdose acts were minor tranquillisers (42% of all, 43% of male and 40% of female overdose acts; P<0.001), paracetamol-containing medication (30% of all, 24% of male and 34% of female overdose acts; P<0.001) and anti-depressants/mood stabilisers (22% of all, 20% of male and 24% of female overdose acts; P<0.001).

Self-cutting was the only other common method of self harm. It was the sole method used in 16% of cases and this was more commonly associated with male (20%) rather than female (13%; P<0.001) presentations. Most cases involving self-cutting were minor and did not require sutures (63% of all, 59% of male and 68% of female cases; P<0.001). However, 6% of cases (8% of male and 4% of female cases; P<0.001) were referred for plastic surgery assessment.

Among men, and to a lesser extent among women, the older the self harm patient, the greater the likelihood that drug overdose was used as the sole method of self harm (Figure 2). The corollary was the relatively higher prevalence of self-cutting among the young self harm patients. The more lethal methods of attempted hanging and drowning were the only means of self harm involved in 3% and 2% of presentations, respectively. It was indicated that alcohol was involved in 41% of all DSH, in 45% of male and 38% of female acts (P<0.001).

### Repeated deliberate self harm

Of the 48,206 people who made the 75,911 DSH presentations to hospital in 2003–2009 that were recorded by the Registry, 10,516 (22%) presented on at least two occasions (at least one repeat), 4,642 (10%) presented at least three times (at least two repeats) and 453 (1%) presented at least ten times. This group of 453 frequent repeaters accounted for 8,080 (11%) of the 75,911 self harm presentations to hospital over the seven years of observation.

### Risk of repetition

**Figure 3:**
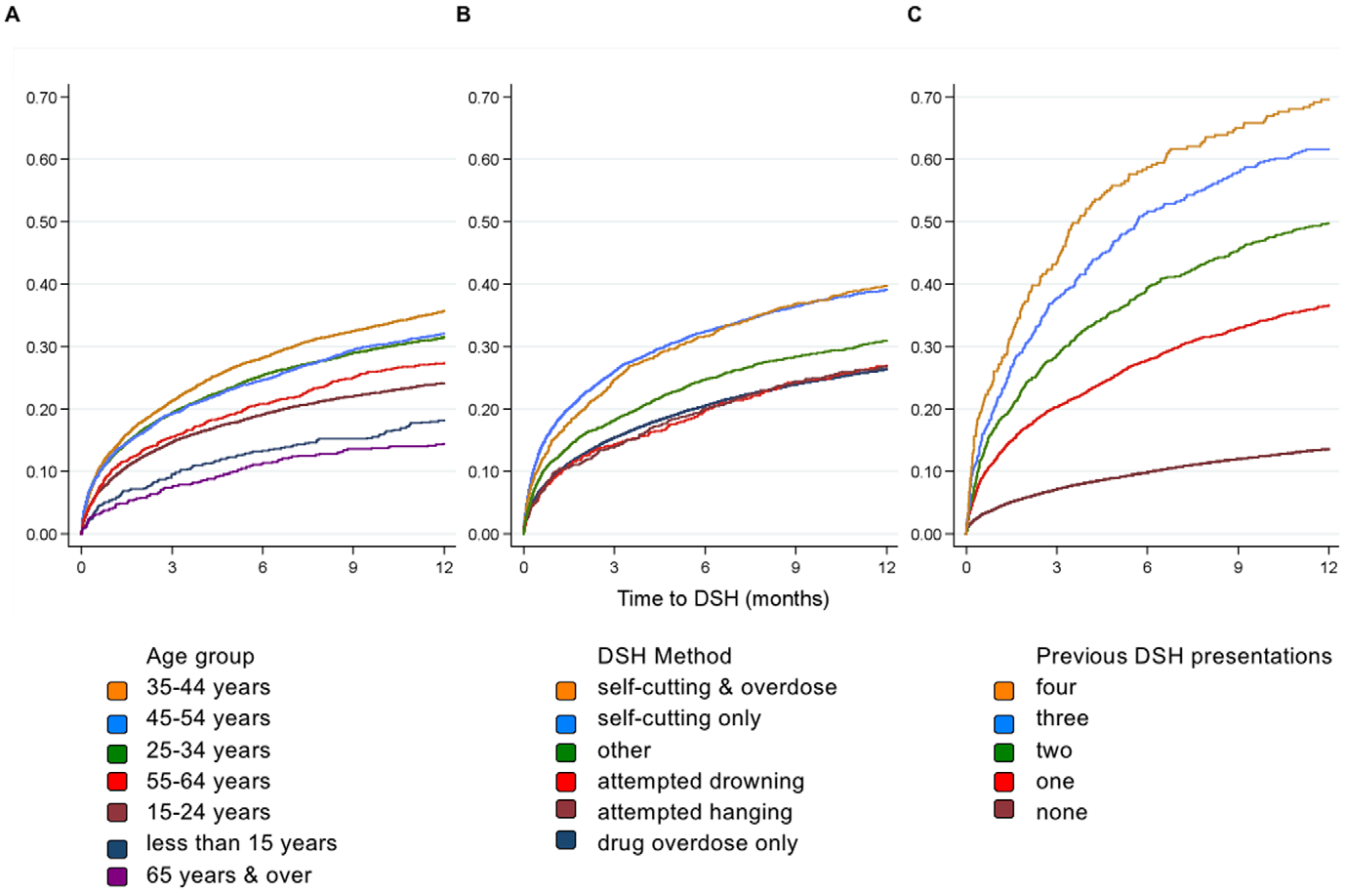
Kaplan-Meier failure curves showing the cumulative probability of a repeated deliberate self harm (DSH) presentation. The curves illustrate the cumulative probability of a repeated deliberate self harm (DSH) presentation to hospital in the twelve month period after an index DSH presentation. Variation in the probability of a repeated DSH presentation is shown by age group (A), by method of DSH (B) and by number of previous DSH presentations.

The results of a Kaplan-Meier analysis are presented in Figure 3. Risk of repetition was highest in the time immediately after a DSH presentation with almost half (45%) of all repeat events occurring in the first 3 months and almost two-thirds (60%) in the first 6 months. Repetition rates were similar in men and women, 29% vs. 30% within 12 months. Risk of repetition within twelve months increased from 18% in those younger than 15 years to 37% in those aged 35–44 years and fell to 14% in those over 65 years. There was a clear association between repetition and the method of DSH with the highest rate observed in patients who used self-cutting, either as the sole method or in combination with drug overdose (Figure 3). Risk of repetition within twelve months of a presentation was 39% and 40% in patients whose methods were cutting only or cutting and overdose, respectively. In contrast, the 12-month risk of repetition for the drug overdose only group was 26% with a similar risk level for other methods. Risk of repetition increased rapidly with the number of previous self harm presentations. The 12-month repetition rate was 14%, 37%, 50%, 62%, and 70% for those with no, one, two, three and four previous presentations.

Table 1 summarises the results of the multivariate Cox regression analysis examining risk of repeated DSH. Results were consistent with univariate results. For both genders the highest repetition rate was in the 35–44 year age group although this was only significant among female patients. Among men, patients aged over 65 years were less likely to make a repeat presentation. Acts involving self-cutting were associated with an elevated risk of repetition for both genders but where cutting was the sole method of DSH the increased risk of repetition among female patients was significantly higher than that among male patients. Risk of repetition increased rapidly with the number of previous DSH presentations and in a similar manner for male and female patients. An alternate analysis which stratified by number of previous DSH presentations yielded similar risk estimates for the age groups and methods of self harm.

**Table 1:** Results of multivariate Cox regression analysis examining 12-month risk of repeated deliberate self harm (DSH).

	Hazard Ratio (95% CI)	Hazard Ratio (95% CI)
	Male	Female
Age group		
<15 years	0.68 (0.41, 1.13)	1.25 (1.00, 1.56)
15 to 24	1.0	1.0
25 to 34	0.98 (0.87, 1.09)	1.20 (1.06, 1.34)
35 to 44	1.12 (0.99, 1.26)	1.34 (1.19, 1.49)
45 to 54	1.01 (0.87, 1.17)	1.15 (1.00, 1.31)
55 to 64	0.87 (0.70, 1.09)	1.12 (0.94, 1.34)
65 and over	0.51 (0.33, 0.79)	0.90 (0.66, 1.21)
Method		
Overdose	1.0	1.00
Cutting	1.18 (1.06, 1.32)	1.57 (1.41, 1.75)
Overdose & Cutting	1.48 (1.23, 1.78)	1.48 (1.23, 1.76)
Hanging	0.96 (0.75, 1.23)	1.21 (0.93, 1.56)
Drowning	0.88 (0.72, 1.09)	1.07 (0.77, 1.47)
Other	1.00 (0.87, 1.15)	1.20 (1.01, 1.42)
Previous DSH presentations		
None	1.0	1.0
One	3.02 (2.72, 3.35)	3.04 (2.76, 3.34)
Two	3.99 (3.48, 4.58)	4.92 (4.33, 5.59)
Three	5.78 (4.89, 6.83)	6.36 (5.38, 7.52)
Four	6.74 (5.24, 8.67)	8.09 (6.71, 9.76)

## Discussion

This study provides the first reliable national-level data on the incidence and repetition of hospital treated self harm. In contrast with suicide, national recording systems for DSH are absent. Previous studies have reported on regional multicentre DSH data within one country [7], [19], [20] or across multiple countries [14], [21], [22] and these have demonstrated striking differences in the incidence of hospital-treated DSH. However, patterns and trends from one centre may be representative of others in the same country as has been demonstrated in the UK [23] where the value of DSH registries has been exemplified, most notably by the Oxford Monitoring System for Attempted Suicide which has contributed hugely to the understanding of suicidal behaviour since its establishment in 1976.

The findings from the Irish National Registry of Deliberate Self Harm highlight the scale of the problem, the extent of variation by age, gender, and the high rates of repeated DSH. The highest DSH rates (700 per 100,000) were observed in 17 year-old girls. Repetition rates were similar in men and women with 29% and 30% engaging in a repeat self harm act in twelve months follow-up, respectively. Risk of repetition is highest in the days and weeks following each presentation.

The findings on the pattern of self harm presentations to hospital by gender, age and method are broadly similar to previous studies in other countries. In particular, the higher rate in women, the peak in early adult life and the predominance of drug overdose as a method of self harm seem to be consistent across difference cultures [24]–[26]. However, rates of self-cutting particularly in men are higher in Ireland than in most other regions in Europe [14]. One previous study also found an over representation of men among self-cutting patients. Compared to overdose patients, they were more often single, lived alone, were unemployed, had a history of previous DSH and misused alcohol [27]. The Irish Registry data show that men inflict more severe damage when self-cutting compared to women which has also been shown elsewhere [28]. This suggests that greater lethality may be the reason for our observed over representation of men among self-cutting patients.

The one-year risk of non-fatal repetition of 29% in men and 30% in women is considerably higher than that estimated from a 2002 systematic review of 90 observational and experimental studies [5] but is similar to the findings of more recent Irish and UK studies which also took an event-based rather than person-based approach to the analysis of repetition [10], [29]. The high rate of repetition in the period immediately following each episode is also consistent with previous work [9]. Risk of DSH repetition rose sharply with increasing numbers of previous self harm presentations. There was a 70% risk of a repeat self harm presentation within twelve months among individuals with four previous presentations. This is in line with Haw et al who found that past history of DSH was the best predictor of both frequent (4+) and less frequent (1–3) repetitions [30].

As with most previous research, there was no reliable clinical record of the total number of previous self harm acts engaged in by the DSH patient who presented to hospital. Therefore, it was not possible to directly estimate the one-year repetition rate of patients who engaged in DSH for the first time, i.e. an inception cohort. However, we feel that our follow-up of patients who presented to hospital for the first time in at least three years will closely approximate the one-year risk of an inception cohort. The high levels of repeated DSH underline the need for suitably-trained health professionals to assess both the risk of repeated suicidal behaviour and the broader psychosocial needs of DSH patients attending hospital EDs and to make appropriate referral and provision for follow-up [31], [32]. However, the provision of assessments to DSH patients has been shown to vary considerably by hospital [33].

In terms of effective treatment interventions reducing risk of repeated self harm, there is a growing evidence base supporting the efficacy of cognitive behaviour therapy, problem-solving therapy and interpersonal psychotherapy [34]–[36]. For borderline personality disorder patients who frequently engage in repeated DSH, dialectical behaviour therapy has consistently shown positive effects in reducing risk of repetition [35]. There is also evidence of efficacy for less intensive interventions as reduced repetition rates have been found among DSH patients who received postcard reminders of the availability of services in the 24 months following discharge from hospital [37], [38].

The increased DSH rate in Irish men in 2008 and 2009 was paralleled by an increase in the male suicide rate and these changes coincided with the advent of the economic recession in Ireland. These findings are in line with Stuckler et al [39] and Gunnell et al [40] and provide further evidence of the effect of recession on rates of suicidal behaviour and the need for preventative action.

The findings from the Irish National Registry of Deliberate Self Harm in relation to repetition underline the importance of linking DSH with suicide mortality data. Patients surviving an act of attempted hanging or attempted drowning showed a relatively low risk of non-fatal repetition. However, studies have shown their risk of fatal repeat acts to be relatively high [41], [42].

In conclusion, the findings from this study highlight the value of a national registry of hospital-treated DSH as a health information system with particular reference to monitoring demographic trends and the identification of predictive factors for repetition and monitoring the health response to this problem.
